# Distance from a Fishing Community Explains Fish Abundance in a No-Take Zone with Weak Compliance

**DOI:** 10.1371/journal.pone.0126098

**Published:** 2015-05-07

**Authors:** Sahir Advani, Laura N. Rix, Danielle M. Aherne, Magdy A. Alwany, David M. Bailey

**Affiliations:** 1 Institute of Biodiversity, Animal Health and Comparative Medicine, University of Glasgow, Glasgow, United Kingdom; 2 Department of Marine Science, Faculty of Science, Suez Canal University, Ismailia, Egypt; Seagrass Ecosystem Research Group, Swansea University, UNITED KINGDOM

## Abstract

There are numerous examples of no-take marine reserves effectively conserving fish stocks within their boundaries. However, no-take reserves can be rendered ineffective and turned into ‘paper parks’ through poor compliance and weak enforcement of reserve regulations. Long-term monitoring is thus essential to assess the effectiveness of marine reserves in meeting conservation and management objectives. This study documents the present state of the 15-year old no-take zone (NTZ) of South El Ghargana within the Nabq Managed Resource Protected Area, South Sinai, Egyptian Red Sea. Previous studies credited willing compliance by the local fishing community for the increased abundances of targeted fish within the designated NTZ boundaries compared to adjacent fished or take-zones. We compared benthic habitat and fish abundance within the NTZ and the adjacent take sites open to fishing, but found no significant effect of the reserve. Instead, the strongest evidence was for a simple negative relationship between fishing pressure and distance from the closest fishing village. The abundance of targeted piscivorous fish increased significantly with increasing distance from the village, while herbivorous fish showed the opposite trend. This gradient was supported by a corresponding negative correlation between the amount of discarded fishing gear observed on the reef and increasing distance from the village. Discarded fishing gear within the NTZ suggested decreased compliance with the no-take regulations. Our findings indicate that due to non-compliance the no-take reserve is no longer functioning effectively, despite its apparent initial successes and instead a gradient of fishing pressure exists with distance from the nearest fishing community.

## Introduction

Fishing is one of the most pervasive anthropogenic activities affecting the marine environment [1,2]. Fishing impacts marine ecosystems by depleting fish populations, altering community structure, degrading habitat through destructive fishing practices, and modifying ecosystem function [3]. No-take marine reserves, areas in which all extractive activities are prohibited, are increasingly being used as tools in conservation and fisheries management [4,5]. The desired outcomes of no-take marine reserves include the restoration of natural community structure and ecosystem function [4,6]. Additionally, reserves often have the explicit aim of supporting adjacent fisheries by encouraging adult emigration (spillover) of target species into fishing grounds and the provision of larvae to support other parts of the population [7,8].

There is widespread empirical evidence that no-take marine reserves on average have higher fish abundance, biomass, size, and species richness than comparable fished areas [9–13]. No-take reserves are more likely to positively affect targeted high-trophic level species, while the responses of non-targeted and lower trophic level species are more variable, with some species not responding to reserve protection or even declining as community structure changes [10,14–16]. Despite the overall documented success of marine reserves, a number of studies have found that due to a lack of enforcement and compliance a substantial number of no-take reserves do not meet their management objectives, effectively rendering them ‘paper parks’ [5,17–20]. Enforcement of reserve regulations or voluntary compliance by the fishing community are critical to the success of no-take reserves in meeting management objectives [12,17,18,21,22]. Poor enforcement and high levels of poaching can severely reduce the positive effects of marine reserves [19,21–24]. However, in the absence of outside enforcement, no-take reserves can still be successful if there is sufficient acceptance by the fishing community and strong community leadership and self-management [21,25,26]. In fact, compliance levels alone can predict fish biomass within reserves [19]. The effects of enforcement and community engagement, or their absence, are not necessarily detectable early in the life of a marine reserve and long-term monitoring programmes of fish populations and continued engagement with fishing communities are essential to evaluate the success of marine reserves in meeting management objectives [12,27].

Nabq Managed Resource Protected Area (MRPA) has a marine area of approximately 122 km^2^ extending 47 km along the western coast of the Gulf of Aqaba (Red Sea), and is part of the Egyptian Environmental Affairs Agency’s system of South Sinai Protectorates [28]. Although development and fishing is restricted within Nabq MRPA, artisanal fishing by the local Bedouin population is permitted using traditional methods [29–31]. The subsistence fishery for herbivorous fish (Scaridae, Siganidae, Acanthuridae) occurs mostly on the reef edge, reef flat and in lagoonal channels using trammel and gill-nets. On the reef slope and deeper lagoons, hook and hand-lines are used to catch predatory fish species (Epinephelidae, Lutjanidae, Lethrinidae) which have a high commercial value [29].The northern 15 km of the MRPA is designated as a scientific reserve, while the remaining coastline is divided into a series of take- and no-take zones with the no-take zones collectively covering approximately 5 km of coastline. These no-take zones, which prohibit all fishing activity, were established in 1995 in consultation with the local Bedouin fisherman to ensure the sustainability of the fishery. This network of small no-take zones was designed to maximise the accessibility of the fished zones to fishermen, as well as to benefit the fishery through the spillover of fish from the no-take zones into these adjacent areas where fishing was allowed [30,32].

Of the five no-take zones, the no-take zone at South El Ghargana is the most extensively studied. It was created in an area that was previously moderately fished and is located between two fished areas or take-zones. The take-zone to the North, El Ghargana, is located opposite the Bedouin village of El Ghargana and is one of the most heavily fished sites in Nabq MRPA due to its ease of access and close proximity to the village. It has relatively high yields and low catch per unit effort compared to the less heavily fished southern take-zone, El Sohop [29,30]. Previous studies performed two, five [30], and seven years [32] after the establishment of the South El Ghargana no-take zone, found that the size and abundance of several species of the targeted predatory fish families, Lethrinidae, Lutjanidae, and Epinephelidae (previously Serranidae), were significantly greater in the no-take zone compared to the surrounding fished areas to the north and south. These studies also provided evidence that the no-take zone was benefiting the adjacent fished areas due to a possible spillover effect from the no-take zone into the adjacent take-zones [32]. Further, an increase in the catch per unit effort (CPUE) in the adjacent take-zones was observed within the first five years of the establishment of the no-take zone [30]. Recently Galal *et al*. [33] found evidence for the continued effectiveness of the overall network of no-take zones within the Nabq MRPA, with higher average abundance of Lethrinids, Lutjanids, and Epinephelids inside no-take zones compared to fished sites. However, this study only compared fish abundance in the NTZ to the heavily fished El Ghargana with no comparison to the southern take-zone, El Sohop. Further, the CPUE for the South Ghargana take-zone had decreased since 2000, attributed to an increase in the number of fishers and non-compliance with fishery regulations [33]. Given the recent reports of non-compliance the aims of the present study were to assess the current state of the NTZ and its effects on fish and coral assemblages. This was achieved through underwater visual census and benthic photography studies within the NTZ and the adjacent designated take areas to the north and south.

## Materials and Methods

### Study area

**Fig 1:**
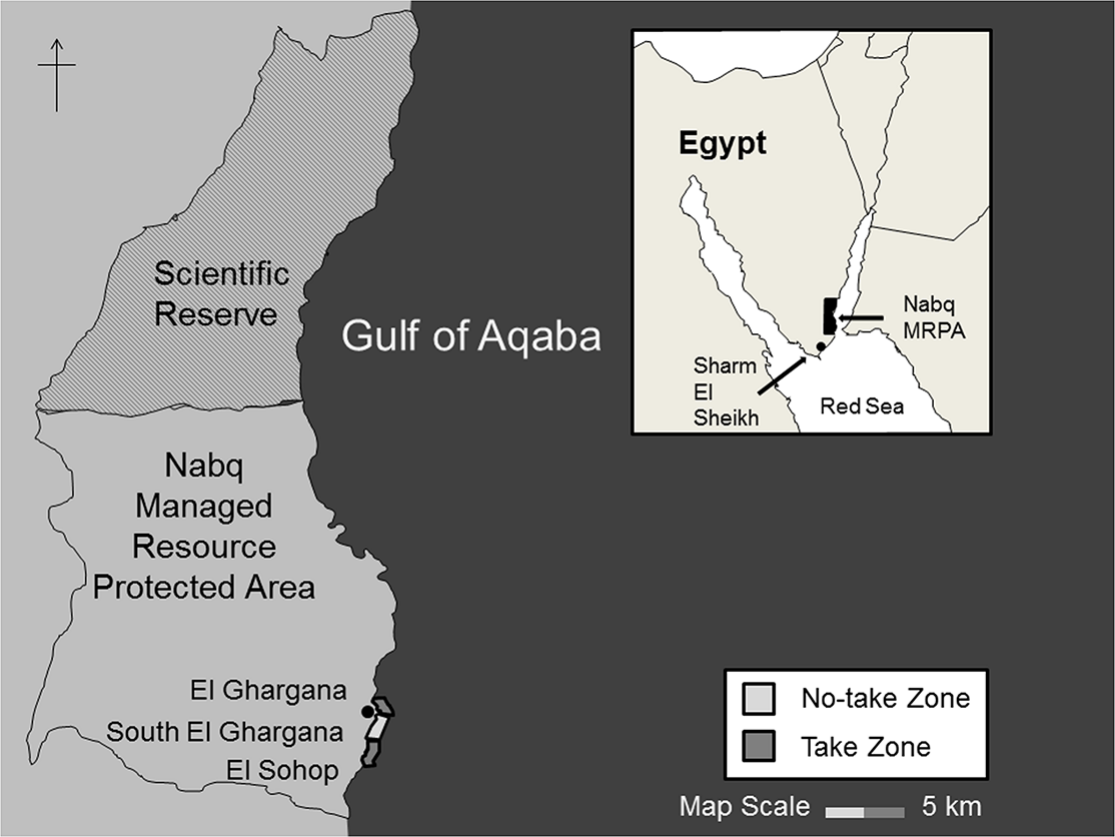
Map showing the location of the Nabq MRPA within Egypt (inset) and the location of the study area within Nabq MRPA. The study area consists of take-zone North (TZ (N)) or El Ghargana, the no-take zone (NTZ) or South El Ghargana, and take-zone South (TZ (S)) or El Sohop. The solid black point marks the village of El Ghargana.

This study focused on the southernmost no-take zone in Nabq MRPA, South El Ghargana, since this is the most extensively studied of the five no-take zones and is located adjacent to the most heavily fished areas in the MRPA [32]. Surveys were conducted inside the 1.2 km no-take zone and up to 1.2 km on either side of the NTZ into the adjacent take areas of El Ghargana to the north and El Sohop to the South (Fig 1). These three zones are herein referred to as the no-take zone (NTZ), take-zone north, TZ (N) and take-zone south, TZ (S). All work was carried out under a permit from the Egyptian Environmental Affairs Agency.

The reef in the study area is a well-developed, semi-continuous fringing reef typical of the Gulf of Aqaba with a broad, shallow reef flat that ranges from 100–800 m in breadth and is broken up in places by small pools and sandy lagoons [29]. At the reef crest, the reef slopes down to a depth that typically ranges between 8 and 12 m before reaching a sandy terrace broken up with coral mounds and seagrass beds [29].

### Survey methods

Fish abundances were estimated using SCUBA Underwater Visual Census (UVC) between June and July, 2011. Within each of the three zones, three sites were haphazardly chosen by selecting the first continuous reef habitat encountered after entry. A gap of approximately 20 m was maintained between zones during site selection to avoid any overlap in sampling. At each site, three 50 m x 5 m belt transects were measured out and marked with floats at 3 and 10 meters depth, for a total of nine transects per zone at each depth. Each transect was surveyed three times on non-consecutive days. In order to avoid pseudo replication these replicates were either averaged to give a single data point or transect location was included as a random factor, depending on the type of analysis. The position of the start and end of each transect was recorded at the surface with GPS. The two depths were chosen to be representative of fish communities at the reef crest and reef slope, respectively [32]. In some cases however, the base of the reef was shallower than 10 m, and at these instances the transect centre line followed the base of the reef to a minimum depth of 7 m.

Three trained observers swam side-by-side 0.5 m above the reef, at a speed of approximately 10 m min^-1^. Fish families were divided between observers with each observer counting a maximum of three morphologically or behaviourally similar families [32]. The use of three observers enabled all fish to be counted in a single pass of the transect. The same three observers performed all transects ensuring any observer bias was equivalent across the study area. Transects were alternated between zones in random order to avoid any effect of practise, with the first transect of the day varying between zones to remove any systematic effect of time of day.

In total 8 families of fish were surveyed; groupers (Epinephelidae), snappers (Lutjanidae), emperors (Lethrinidae), butterflyfishes (Chaetodontidae), angelfishes (Pomacanthidae), parrotfishes (Scaridae), surgeonfishes (Acanthuridae), and rabbitfishes (Siganidae). These families included targeted and non-targeted species with the families Scaridae, Siganidae, Acanthuridae, Lethrinidae, and Epinephelidae comprising the highest proportion of the local fishery’s catch [29]. All fish were identified to the species level except for Scaridae, as species from this family could not always be distinguished. For trophic level analysis, species were divided into five trophic categories; piscivore, invertivore, corallivore, omnivore, and herbivore, based on their trophic score and feeding behaviour determined from FishBase ([34], see Table 1). In this way species from the same family were sometimes classified into different trophic groups.

**Table 1:** Species surveyed during the study, the trophic category to which they were assigned and each category’s average trophic level.

Trophic Category	Average Trophic Level	List of Species
Herbivore	2.07	*Acanthurus nigrofuscus*, *A*. *sohal*, *Ctenochaetus striatus*, *Naso elegans*, *N*. *unicornis*, *Zebrasoma desjardinii*, *Z*. *xanthurum*, *Hipposcarus harid*, *Scarus niger*, *Siganus argenteus*, *S*. *luridus*, *S*. *stellatus*
Omnivore	2.95	*Chaetodon paucifasciatus*, *C*. *auriga*, *C*. *fasciatus*, *Pomacanthus maculosus*, *P*. *imperator*, *Pygoplites diacanthus*
Corallivore	3.34	*Chaetodon austriacus*, *C*. *lineolatus*, *C*. *melannotus*, *C*. *trifascialis*
Invertivore	3.44	*Heniochus intermedius*, *Chaetodon semilarvatus*, *Monotaxis grandoculis*, *Lethrinus nebulosus*, *L*. *obsoletus*, *L*. *mahsena*, *L*. *borbonicus*, *L*. *harak*, *Epinephelus*. *fasciatus*,
Piscivore	4.21	*Lutjanus bohar*, *L*. *monostigma*, *L*. *ehrenbergi*, *Macolor niger*, *Cephalopholis argus*, *C*. *hemistiktos*, *C*. *miniata*, *Epinephelus malabaricus*, *E*. *tauvina*, *Plectropomus pessuliferus*, *Variola louti*

The presence of discarded fishing gear was recorded as it can be used to estimate fishing activity as well as non-compliance in and around marine reserves [32,35]. Discarded fishing gear was recorded on the reef slope at each transect and consisted of hook and hand-lines and fishing nets. Each individual item was counted (e.g. a continuous section of line with multiple hooks being a single item) and no differentiation was made between the type of gear. Data were pooled to give the total number of discarded fishing gear items per zone. These data are not intended to provide a quantitative measurement of fishing effort or non-compliance, but rather a qualitative estimate of the relative fishing pressure and compliance levels in the different zones assuming that fishing gear is lost at an equal rate across zones depending on how much fishing is occurring.

### Video transects of benthic substrate

To estimate differences in benthic substrates between zones, one 50 m transect from each site was videoed at both 3 and 10 m. The video camera (Canon PowerShot G7, Canon U.S.A, Inc.) was held vertically approximately 40–50 cm from the substrate in order to give a field of view of approximately 0.5 m^2^, and swimming speed was kept constant at 10 m min^-1^.

Still frames were then captured from the video using VideoPad Video Editor Professional v. 2.41 software [36] every 3 seconds, approximately corresponding to a distance of 0.5 m between frames, for a total of 100 still frames per 50 m video transect. Still frames were imported into the software package “Coral Point Counts with Excel extensions” CPCe v. 4.0 [37], and for each frame CPCe randomly selected five points for identification. The substrate categories used for identification were hard coral, soft coral, coralline algae, algal turf, macroalgae, old dead coral, recently dead coral, and rock/sand/rubble.

### Statistical analysis

#### Trophic group and family abundance

Generalized linear mixed models (GLMMs) with a Poisson distribution were used to analyse differences in abundances of individual fish families and trophic groups across the three zones using the lme4 package [38] in the statistical software R v.2.15.1 [39]. To account for the repeated sampling of transects and temporal variation, both transect replicate and the sampling date were included as random factors in the model. Zone was a fixed factor and the significance of zone was determined after iteratively removing factors from the model and using log-likelihood ratio tests to examine how well the model fit. The model with the best fit was

[fish family / trophic group]∼zone+(1|sampling_date)+(1|transect_replicate)

Plots of residuals were visually checked to ensure normality and homogeneity of variance. No instances of overdispersion were observed, nor was zero-inflation a concern. If zone was found to be significant overall, Tukey multiple comparison tests with Bonferroni adjustment were used to determine specifically which pairs of zones exhibited significant differences. This was accomplished using the multcomp package [40] in R v.2.15.1.

In addition to testing for significant differences between the three distinct zones, a similar GLMM model was constructed replacing zone with distance as the fixed factor to examine the relationship between trophic group abundances and the distance from the village of El Ghargana at the northern end of the study area. Since TZ (N) was directly in front of the village, all transects in TZ (N) were considered to be at 0 m, and the distance of all further transects from the edge of the village was measured using the GPS coordinates for each transect.

#### Fish community structure

Multivariate analysis of variation in the fish community between zones was accomplished in the statistical software PRIMER-E (Plymouth Routines In Multivariate Ecological Research) v. 6.l.10 [41]. For fish community analysis, the three temporal replicates of each 50 m transect were averaged to produce a single data point. Species abundance data were square root transformed to allow both common and rare species to contribute to the determination of ranked similarities and a similarity matrix was created using Bray-Curtis coefficients. Species level variation in the fish community across zones at both depths was visualised using two-dimensional non-metric Multi-Dimensional Scaling (MDS) plots, with stress values less than 0.2 considered a good representation of the community data. The significance of these differences was then tested using a two-way Analysis of Similarities (ANOSIM) with values less than 0.05 considered significant. ANOSIM pair-wise tests were used to identify which pairs of zones were significantly different and their degree of dissimilarity.

#### Discarded fishing gear

Differences in the total number of discarded fishing gear items was analysed between zones and as a function of distance from the village. Kruskal Wallis Multiple Comparison tests were used to analyse differences between zones and a one-tailed Spearman’s Rank Correlation test was used to determine the relationship between the quantity of discarded fishing gear and distance from the village, as these data did not meet the assumptions for parametric testing.

#### Benthic substrate

Benthic community data was analysed in PRIMER v.6.1.10 [41]. Data were first log(x+1) transformed to allow both common and rare substrate types to contribute to the determination of ranked similarities. The difference in the benthic community between zones was tested using a one-way ANOSIM. Differences in the percent cover of individual substrates across zones were tested for significance using Kruskal Wallis tests, as data did not meet assumptions for parametric testing. Percent cover data were arcsine transformed [42].

## Results

### Family abundance

**Fig 2:**
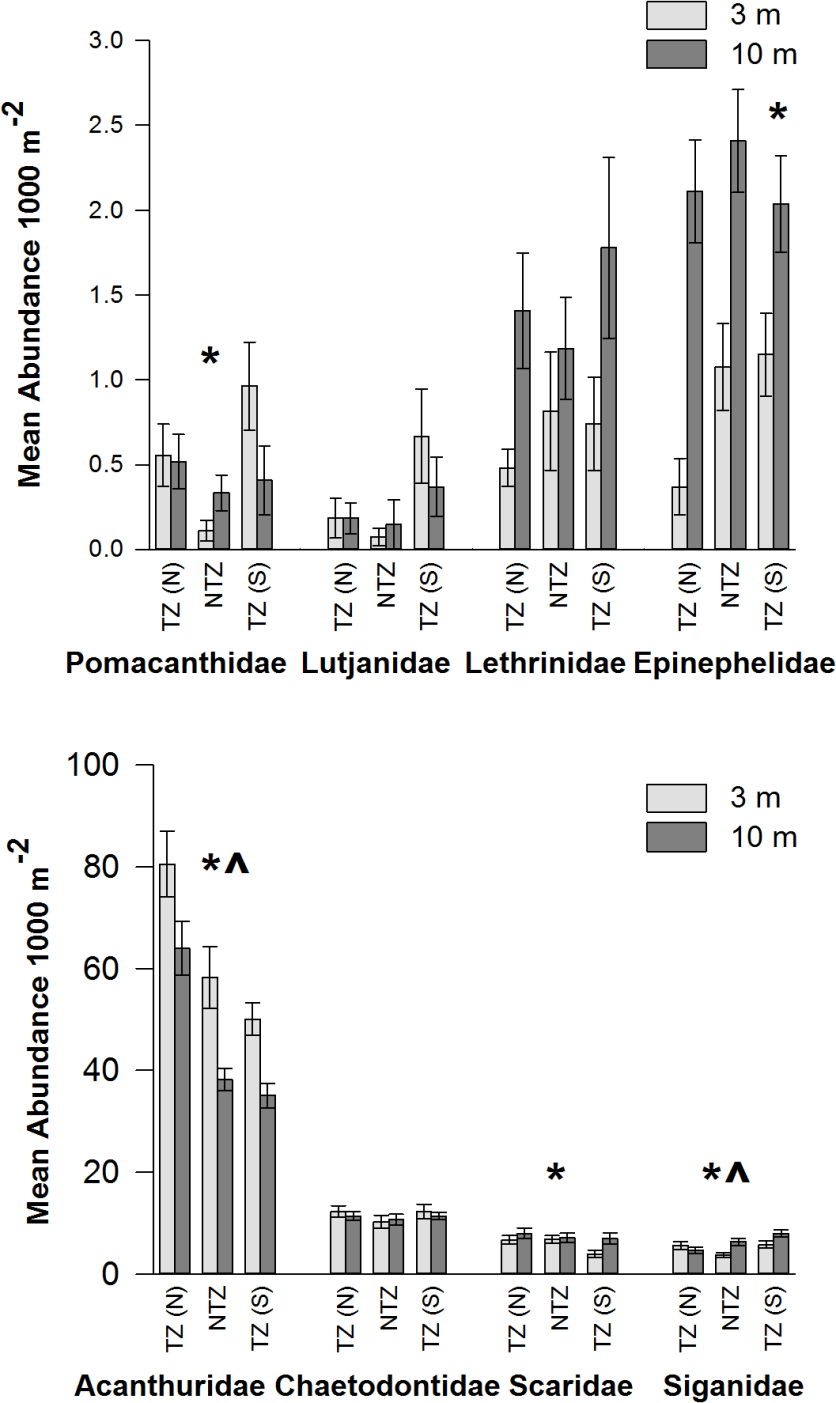
Mean abundance (1000 m^-2^ ± SE) of fish families at 3 and 10 m across the three zones; take-zone North TZ (N), the no-take zone NTZ, and take-zone South TZ (S). * indicates significant differences between zones at 3 m. ^ indicates significant differences between zones at 10 m.

There was no clear trend in fish abundance across zones for the different fish families (Fig 2). However, significant differences were most frequent between TZ (N) and the NTZ and/or TZ (S), with few between NTZ and TZ (S) (Table 2). Epinephelidae were significantly more abundant in both TZ (S) and NTZ compared to TZ (N) at 3 m depth. Acanthuridae showed the opposite trend and were significantly more abundant in TZ (N) compared to both NTZ and TZ (S) 3 m (Table 2). Only Pomacanthidae and Siganidae exhibited significant differences between TZ (S) and NTZ at 3 m, showing higher abundances in TZ (S) (Table 2). Overall zone was a significant factor in explaining Scaridae abundance, but multiple comparison tests revealed no individual pairs of zones were significantly different. Only two families displayed significant differences at 10 m depth, Siganidae were significantly more abundant in TZ (S) compared to TZ (N) and Acanthuridae showed the same trend as at 3 m with significantly higher abundances in TZ (N) compared to both NTZ and TZ (S). The non-targeted family Chaetodontidae showed a very uniform distribution across zones at both depths (Fig 2). None of the families were significantly more abundant in the NTZ compared to both take-zones.

**Table 2:** Results of Generalized Linear Mixed Models (GLMM) of differences in abundances of fish families at 3 and 10 m between the zones with post-hoc Bonferroni corrected Tukey pairwise multiple comparisons.

Family	Zone^a^	TZ (N)—NTZ^b^	NTZ—TZ (S) ^b^	TZ (N)—TZ (S) ^b^
**3 m**				
**Acanthuridae**	**10.515, 2, 0.005**	**-2.911, 0.011**	-0.396, 1	**-3.312, 0.003**
**Chaetodontidae**	1.919, 2, 0.383	-	-	-
**Lethrinidae**	0.339, 2, 0.844	-	-	-
**Lutjanidae**	4.257, 2, 0.119	-	-	-
**Pomacanthidae**	**11.373, 2, 0.003**	2.094, 0.109	**3.001, 0.008**	1.290, 0.591
**Scaridae**	**6.321, 2, 0.042**	-0.053, 1	-2.386, 0.051	-2.306, 0.063
**Epinephelidae**	**7.570, 2, 0.023**	**-2.411, 0.048**	0.092, 1	**2.493, 0.038**
**Siganidae**	**8.016, 2, 0.018**	**2.749, 0.018**	**2.638, 0.025**	-0.151, 1
**10 m**				
**Acanthuridae**	**31.575, 2, <0.001**	**6.230, <0.001**	-1.041, 0.894	**-7.254, <0.001**
**Chaetodontidae**	0.486, 2, 0.785	-	-	-
**Lethrinidae**	0.157, 2, 0.925	-	-	-
**Lutjanidae**	1.911, 2, 0.385	-	-	-
**Pomacanthidae**	0.816, 2, 0.665	-	-	-
**Scaridae**	0.681, 2, 0.711	-	-	-
**Epinephelidae**	0.668, 2, 0.716	-	-	-
**Siganidae**	**6.394, 2, 0.041**	-0.450, 0.441	1.254, 0.629	**2.687, 0.022**

Results for individual pairs of zones are shown only when the overall difference between zones is significant.

^a^ is Chi-square statistic, degrees of freedom, and *p* value for the GLMM.

^b^ is the Chi-square statistic and *p* value for Bonferroni corrected pairwise multiple comparison test.

### Trophic level abundance

**Fig 3:**
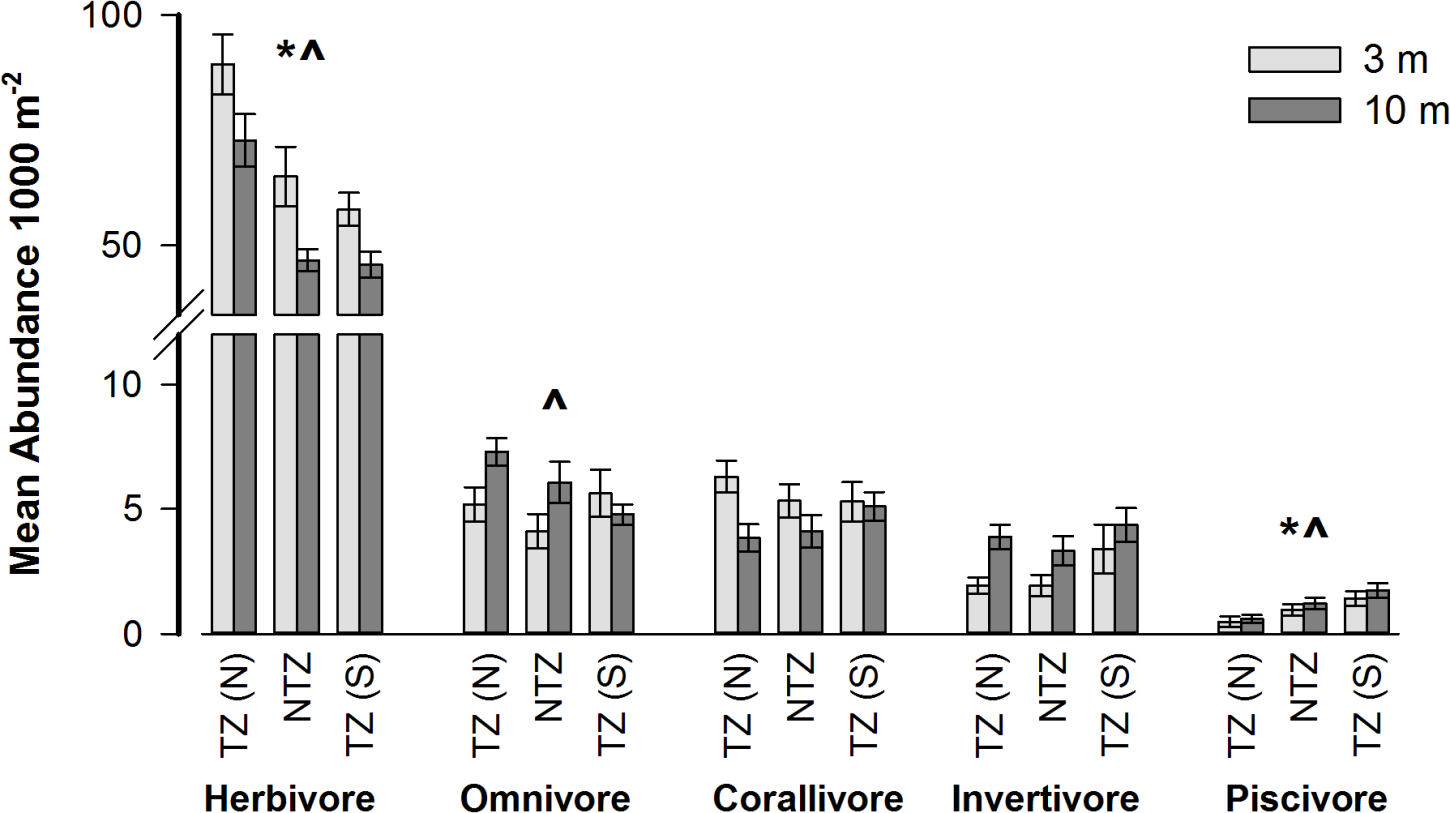
Mean trophic level abundance (1000 m^-2^ ± SE) at 3 and 10 m across the three zones; take-zone North TZ (N), the no-take zone NTZ, and take-zone South TZ (S). * indicates significant differences between zones at 3 m. ^ indicates significant differences between zones at 10 m.

When species were grouped by trophic level, piscivores increased from take-zone north to take-zone south at 3 and 10 m with significantly greater abundances in TZ (S) than TZ (N) at both depths (Table 3, Fig 3). Herbivores showed the opposite trend, with decreasing abundance from take-zone north to take-zone south at both 3 and 10 m and at both depths were significantly more abundant in TZ (N) compared to both NTZ and TZ (S) (Table 3). Omnivores were significantly more abundant in TZ (N) compared to TZ (S) at 10 m but showed no observable trend at 3 m (Table 3). Neither corallivores nor invertivores showed any significant differences in abundances across zones (Table 3).

**Table 3:** Results of Generalized Linear Mixed Models (GLMM) of differences in abundances of trophic levels at 3 and 10 m between the zones with post-hoc Bonferroni corrected Tukey pairwise multiple comparisons.

Trophic Level	Zone^a^	TZ (N)—NTZ^b^	NTZ—TZ (S) ^b^	TZ (N)—TZ (S) ^b^
**3 m**				
**Herbivore**	**10.913, 2, 0.004**	**3.068, 0.006**	-0.240, 1	**-3.315, 0.003**
**Omnivore**	2.145, 2, 0.342	-	-	-
**Corallivore**	1.194, 2, 0.551	-	-	-
**Invertivore**	2.421, 2, 0.298	-	-	-
**Piscivore**	**6.509, 2, 0.039**	-1.673, 0.283	0.988, 0.990	**2.585, 0.029**
**10 m**				
**Herbivore**	**26.974, 2, <0.001**	**5.818, <0.001**	-0.386, 1	**-6.203, <0.001**
**Omnivore**	**6.353, 2, 0.042**	1.271, 0.611	-1.455, 0.437	**-2.713, 0.020**
**Corallivore**	2.163, 2, 0.339	-	-	-
**Invertivore**	0.942, 2, 0.624	-	-	-
**Piscivore**	**13.058, 2, 0.001**	-2.350, 0.056	1.552, 0.362	**3.687, <0.001**

Results for individual pairs of zones are shown only when the overall difference between zones is significant.

^a^ is Chi-square statistic, degrees of freedom, and *p* value for the GLMM.

^b^ is the Chi-square statistic and *p* value for Bonferroni corrected pairwise multiple comparison test.

The relationship between distance from El Ghargana village at the north end of the study area and the mean abundance of each trophic group is consistent with the above findings. At both depths, piscivores increased significantly in abundance with increasing distance from the village (Chi-sq = 6.540, p = 0.011 at 3 m; Chi-sq = 13.058, p <0.001 at 10 m), while herbivores decreased significantly over the same distance (Chi-sq = 11.011, p <0.001 at 3 m; Chi-sq = 13.437, p <0.001 at 10 m). Other trophic levels did not exhibit any significant differences in abundances as a function of distance, but instead showed relatively uniform distributions across the study area.

### Fish community structure

**Fig 4:**
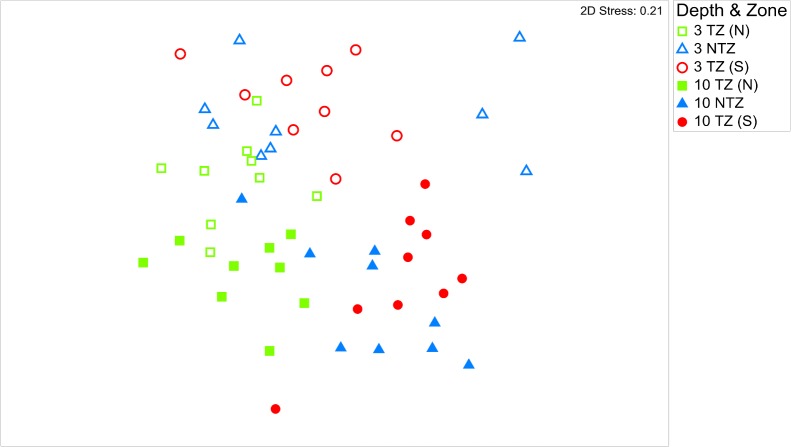
2-D MDS ordination of the fish assemblage at 3 m and 10 m across the three zones; take-zone North TZ (N), the no-take zone NTZ, and take-zone South TZ (S).

The MDS plot revealed that the no-take zone did not cluster separately from the designated take zones (Fig 4). While some overlap occurs between all three zones, the greatest separation appears to be between TZ (N) and TZ (S), while the NTZ is intermediate between the two (Fig 4), though it should be noted that the stress value of the MDS plot of 0.21 slightly exceeded the threshold of 0.2. The ANOSIM test revealed that there were significant differences between all zones, however, the greatest dissimilarity occurred between take-zone north and take-zone south (R = 0.501, p = 0.001), and the least dissimilarity occurred between the no-take zone and take-zone south (R = 0.136, p = 0.006) (Table 4). Additionally, the two depths clustered separately indicating there were differences in community structure between 3 and 10 m (Fig 4).

**Table 4:** ANOSIM pairwise tests for differences in the fish community between pairs of zones at both depths of 3 and 10 m.

Pairs	Observed R Statistic	Significance Level	Possible Permutations	Actual Permutations	Permutations where R ≥ Observed R
NTZ, TZ (N)	0.286	0.001	590976100	999	0
NTZ, TS (S)	0.136	0.006	590976100	999	54
TZ (N), TZ (S)	0.501	0.001	590976100	999	0

### Discarded fishing gear

**Fig 5:**
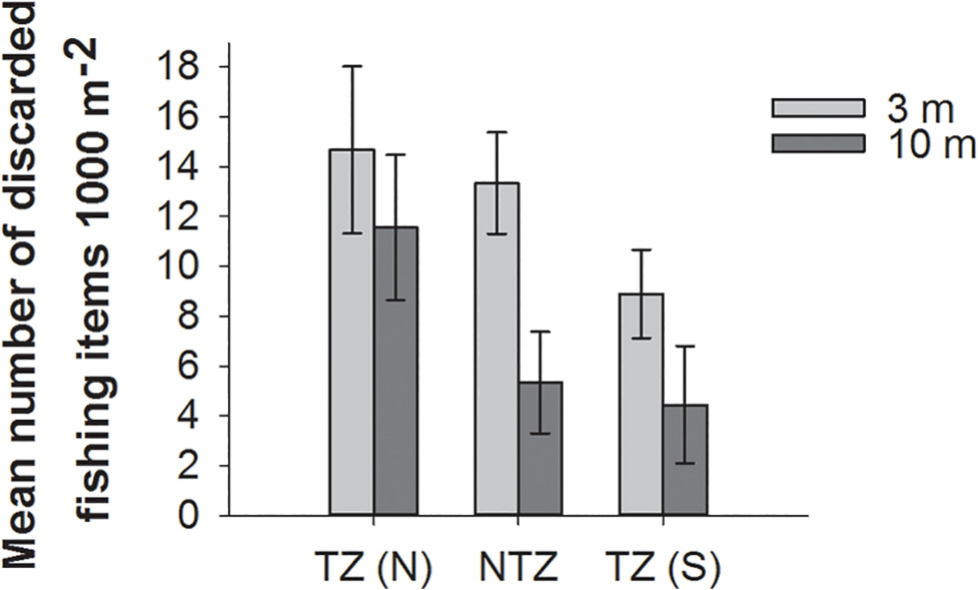
Mean number of fishing gear items (1000 m^-2^ ± SE) recorded within each of the three zones; take-zone North TZ (N), the no-take zone NTZ, and take-zone South TZ (S).

Discarded fishing gear consisted primarily of hook and hand-line and to a lesser extent gill and trammel nets and were significantly more abundant at 3 m than at 10 m (Kruskal Wallis rank sum test, *H* = 5.7435, df = 1, p = 0.0166). Discarded fishing gear items were also most abundant in take-zone north and least abundant in take-zone south at both depths (Fig 5), however, this difference was not significant at either 3 or 10 m (*H* = 2.5747, df = 2, p = 0.276 and *H* = 3.4124, df = 2, p = 0.1816, respectively). However, when discarded fishing gear was plotted as a function of distance from the village of El Ghargana the observed fishing gear decreased linearly from north to south across the study area at both depths and this negative correlation was significant at both 3 and 10 m (Spearman’s rank correlation, *r* = -0.655, p = 0.028 and *r* = -0.761, p = 0.009, respectively).

### Benthic substrate

Benthic community composition did not differ significantly between the take and no-take zones at either 3 or 10 m (R = 0.085, p = 0.184 and R = 0.148, p = 0.085, respectively). In particular, the percent cover of individual substrates (e.g. hard coral, soft coral, recently dead coral, and algae) did not vary significantly across zones (Kruskal Wallis tests, p > 0.05). Live hard coral cover ranged from 34.7 ±3.4% to 37.5 ±8.2% across zones at 3 m, and 23.6 ±2.9% to 26.3 ±2.1% at 10 m.

## Discussion

Significant differences in fish abundances and community structure were found between the no-take and take-zones, but these differences were greatest between the two take-zones (TZ (N) vs. TZ (S)), while the central no-take zone (NTZ) was typically intermediate in abundance and species composition. These results are in stark contrast with the expectations for a functional no-take zone, especially the prediction that there would be greater abundances of targeted species in the no-take zone compared to both adjacent take-zones [4,10,43]. This also contrasts with the results of previous studies that found increased abundances of targeted predatory fish in the NTZ compared to both take-zones [30,32]. These findings indicate that the South El Ghargana no-take zone is no longer providing adequate protection to the fish community within its boundaries and can now be regarded as a paper park.

Data at the family level provided little evidence of a protective effect within the NTZ as no fish families showed significantly higher abundances in the NTZ compared to both take-zones. The differences between zones were most apparent once the species had been grouped by trophic level. Since no-take reserves can impact different trophic levels in different ways, grouping species by functional groups may give clearer trends than analyzing groupings by families, which can encompass multiple trophic levels [10]. As a key example, the most valuable food fish are all piscivores and are particular targets of fishing using hooks and hand-lines in this area [29]. Piscivores had the highest abundance in TZ (S) and only showed a significant difference in abundance between TZ (S) and TZ (N). These high trophic species are an important fisheries target and with increasing fishing pressure decline rapidly. Consequently, studies have consistently shown these fish to provide the greatest positive responses to reserve protection, with typically higher abundances inside reserves in comparison to fished sites outside reserves [10,14,43,44]. However, the abundance patterns observed in this study indicate that fishing pressure on piscivores is highest in TZ (N), intermediate in the NTZ and lowest in TZ (S). This finding is consistent with the observed trends in discarded fishing gear (Fig 5), but contrasts with previous findings that found the least amount of fishing gear and highest abundance of predatory fish (including Epinephelidae) in the no-take zone compared to the adjacent take-zones [32].

Despite all herbivorous families (Scarids, Acanthurids, Siganids) also being targeted by the fishery [29], herbivores showed the opposite trend to that displayed by piscivores, with the greatest abundance in TZ (N) and least in TZ (S) and significantly higher abundances in both TZ (N) and NTZ compared to TZ (S). This contradicted the discarded fishing gear data suggesting herbivores were not directly responding to fishing pressure. However, the discarded gear consisted mainly of hooks and hand-lines, while herbivores are caught primarily with nets on the reef flat. The preferred locations for fishing by hook and hand-line versus nets can differ, which could explain this difference [29]. However, the increase in total herbivore numbers in the zones where piscivores are least abundant (TZ (N) and NTZ) could also be the result of decreased predation in areas where high fishing pressure has depleted piscivore populations (predator release) [3,45]. Herbivore abundance appeared to be driven by the highly abundant Acanthuridae family. In the Red Sea, piscivorous fish, such as groupers, are known to prey heavily on some herbivores, particularly small acanthurids [46]. Such interactions have been suggested to explain the higher abundance of herbivorous families in fished compared to non-fished areas in South El Ghargana and elsewhere in the Red Sea [4,32]. Omnivores were also found to be significantly more abundant in TZ (N) compared to TZ (S) at 10m only while other trophic groups were not significantly different across zones. The varying responses of these different trophic groups is consistent with a number of other studies which have demonstrated that not all species or trophic groups respond equally to fishing pressure or reserve protection [10,11,14]. While the present study utilizes density estimates of fish species, it is important to note that in certain instances estimates of biomass are more effective method for measuring reserve success [11–13]. Within functional and old reserves densities of predatory fish could reach maximum thresholds after a certain point, while biomass would continue increasing [11]. Estimates of biomass would have accounted for this factor in our study, however, maximum fish species density thresholds have probably not been reached in the NTZ given the higher abundance of piscivores in TZ (S).

Results of the multivariate analysis of fish community structure further support the above findings that there were no clear differences between the designated ‘NTZ’ and the two adjacent take-zones. While there are significant differences between the three zones, the greatest differences are between TZ (N) and TZ (S), whereas the NTZ appears to be intermediate between the two. This coincides with the findings of other studies that found that sites with low protection cluster closely to unprotected sites [24]. By contrast, MDS plots of reserves with long-term and effective enforcement of no-take regulations, such as in Apo, Philippines [8] and in Maria Island and Tinderbox, Tasmania [27], feature reserve sites clustering distinctly away from fished reference sites. Thus our results are not in accordance with the pattern observed in long-term, effectively enforced sites. However, we do find significant differences between depths, which is consistent with the higher fishing pressure at 3m compared to 10m water depth in the Nabq MRPA [30,32].

Collectively, the results of the family, trophic level, and community structure data, provide little support for the no-take zone being as effective as previously reported. While there still appears to be greater abundance of some targeted fish in the NTZ compared to the heavily fished TZ (N), higher abundance of some targeted fish, in particular piscivores, were observed in TZ (S) compared to NTZ. This suggests that the changes in abundance are occurring primarily from north to south. In fact, when abundance was plotted as a function of distance from El Ghargana village, distance was found to have a significant effect on the abundance of two trophic groups. Piscivores showed a significant linear increase in abundance from north to south across the study area while herbivores significantly decreased across the same distance. The amount of discarded fishing gear recorded also showed a significant negative linear relationship with distance from the village. Thus, fish abundance appears to be responding to a gradient of decreasing fishing pressure from north to south across the study area rather than a strong no-take reserve effect. This gradient resembles the patterns in fishing intensity that existed in the study area before the establishment of the NTZ i.e. highest in TZ (N) and least in TZ (S) [29]. Thus the results of this study are consistent with the abundance patterns expected in a scenario of diminished reserve effect and indicate a change in compliance compared to earlier studies [32]. It should be noted that there were methodological differences compared to previous studies in terms of the length and width of the transects surveyed and how the data was analyzed. However, the current study covered a subset of the same area as previous studies and despite the methodological differences, the authors believe the transects surveyed are representative of the study area and that the methodologies used by this and previous studies are comparable in terms of relative fish abundances between zones. The recent study by Galal *et al*. [33] describes a 20% reduction in CPUE in the larger network of Nabq MRPA’s take and no-take areas in the 15 years since establishment. The authors attribute this to increased fishing pressure and non-compliance of reserve regulations by fishers. Our present study, with its focus on only South El Ghargana NTZ and its adjacent designated take areas, supports this particular scenario.

The reduced reserve effect in the NTZ and observations of fishing gear within its boundaries along with multiple instances of illegal fishing occurring in the NTZ (S. Advani, pers. obs.), suggest that notable levels of poaching are occurring. Indeed, Galal *et al*. [33] found that South Ghargana had some of the highest levels of poaching of the Nabq NTZs. Poaching could explain the lower than expected abundances of high trophic level fish in the NTZ, as even low levels of poaching can significantly reduce the beneficial effects of no-take reserves, particularly as poaching in NTZs may have high catch rates and preferentially target large, upper trophic level fish [19,23,47]. A number of factors can reduce instances of poaching in no-take reserves, such as effective enforcement measures and clear demarcation of reserve boundaries [21]. The boundaries of South El Ghargana are poorly marked with only one ambiguous sign marking the boundary some distance inland from the shore, and this may encourage encroachment into the NTZ. Additionally, a recent study on the status of marine protected areas in Egypt highlights the limited resources available to the park rangers responsible for enforcing regulations in Nabq [31].

The evidence of poaching in South El Ghargana is disappointing given previous findings that reported on its effectiveness as a reserve and the support it received from the fishing community at the time of its creation [30]. The community was involved in the design of the reserves and made aware of the potential ecological and fisheries benefits of marine reserves through a series of education and awareness campaigns. In addition, a system of self-enforcement via “community rangers” was in place to ensure adequate enforcement of the no-take regulations [30]. Community support and the perception that the fishery benefits from the reserve are factors that have been found to increase the likelihood of compliance and reserve success [13,17–21,25]. Community-driven or opportunistic site selection have been shown to be beneficial for reserve establishment and conservation planning [48,49]. In the case of the El Ghargana fishing community, proximity to the no-take zone may have caused compliance to erode over time. When potential sites for establishing no-take zones were being selected, the local community showed a preference for sites that were infrequently fished or difficult to access. South El Ghargana, a moderately fished site adjacent to a fishing village, was included in Nabq’s network of no-take reserves in order to provide the most useful demonstration of a reserve effect, rather than fisher preference [29].

New findings indicate that isolation and a large size are important features for marine reserves to be successful [12]. The network of five small no-take zones in the Nabq MRPA were systematically established in order to increase spillover and sustain the local fishery [30]. Fifteen years on, documented decreases in CPUE in the larger network of no-take areas in Nabq [33], coupled with our findings of reduced reserve effect and increased non-compliance in the South El Ghargana no-take zone suggest that the initial design of the Nabq MRPA may have been flawed. A single large no-take reserve isolated from local fishing effort may have promoted compliance and provided better protection for reef fish stocks in the region.

Habitat may confound the interpretation of results of reserve studies, as differences in habitat can influence fish abundances [50–52]. In the present study there were no significant differences in the cover of individual substratum types, or in their relative frequencies. Together with a relatively uniform distribution of non-targeted corallivorous fish, these findings indicate that current fishing practices may not be having a substantial impact on the coral reef habitat in Nabq. The fishing intensity in Nabq, even in the most heavily fished areas, is believed to be only moderate, and in addition to the no-take zones, the artisanal fishery is further regulated by gear restrictions such as minimum mesh sizes and fishing practices such as dynamite fishing, spearfishing, and trawling are prohibited.

## Conclusion

This study indicates that the designated no-take zone is having little or no effect on fishing pressure or its ecological consequences and is now functioning as a ‘paper park’. Instead, a gradient of decreasing fishing pressure with increasing distance from El Ghargana village is the simplest explanation for the results obtained. These results are in clear contrast to the results of previous studies and are disappointing given the apparent level of community involvement in setting up the NTZs, as well as the apparent benefits to the fishing community from the NTZ. On a more positive note, the evidence that current fishing practices do not appear to be significantly impacting the benthic habitat, and reports that other NTZs in the area remain operational [33] are a source of optimism that with improved engagement and enforcement the situation in Nabq can be resolved to the satisfaction of all parties. Even in their currently reduced state it appears that fish biomass in the four Nabq NTZs is still higher on average than prior to closure [33]. The results demonstrate that in order to achieve fisheries management and conservation objectives, long-term efforts are required to monitor the state of NTZs and that continued work and dialogue with the affected human community is needed.

## Supporting Information

S1 Dataset Data used in Family level analysis. (XLSX)

S2 Dataset Data used in Trophic level analysis. (XLSX)

S1 Table Mean abundance (1000 m-2 ± SE) of fish families at 3 and 10 m across the three zones; take-zone North TZ (N), the no-take zone NTZ, and take-zone South TZ (S). Data corresponds to Fig 2.
(XLSX)

S2 Table Mean trophic level abundance (1000 m-2 ± SE) at 3 and 10 m across the three zones; take-zone North TZ (N), the no-take zone NTZ, and take-zone South TZ (S). Data corresponds to Fig 3.
(XLSX)

S3 Table ANOSIM pairwise tests for differences in the fish community between pairs of zones at depths of 3 m, 10 m and both depths. (XLSX)

S4 Table Mean number of fishing gear items (1000 m-2 ± SE) recorded within each of the three zones; take-zone North TZ (N), the no-take zone NTZ, and take-zone South TZ (S). Data corresponds to Fig 5.
(XLSX)
